# Cross-Sectional Comparative Study on Central Sensitization-Psychosocial Associated Comorbidities and Psychological Characteristics in Breast Cancer Survivors with Nociceptive Pain and Pain with Neuropathic Features and without Pain

**DOI:** 10.3390/life12091328

**Published:** 2022-08-27

**Authors:** Irene de la Rosa-Díaz, Laura Barrero-Santiago, Paz Acosta-Ramírez, Marina Martín-Peces-Barba, Esther Iglesias-Hernández, Bastien Plisset, Nicolás Lutinier, Margot Belzanne, Roy La Touche, Mónica Grande-Alonso

**Affiliations:** 1 Departamento de Fisioterapia, Centro Superior de Estudios Universitarios La Salle, Universidad Autónoma de Madrid, Aravaca, Calle Ganímedes, n° 11, 28023 Madrid, Spain; 2 Physical Therapist in Women’s Health Research Group, Department of Physical Therapy, University of Alcalá, Avenida de León, 3A, 28805 Madrid, Spain; 3 Motion in Brains Research Group, Institute of Neuroscience and Sciences of the Movement (INCIMOV), Departamento de Fisioterapia, Centro Superior de Estudios Universitarios La Salle, Universidad Autónoma de Madrid, Aravaca, Calle Ganímedes, n° 11, 28023 Madrid, Spain; 4 Physical Therapy in Torrejón Hospital, Rehabilitation Department, Calle Mateo Inurria, 28850 Madrid, Spain; 5 Instituto de Dolor Craneofacial y Neuromusculoesquelético (INDCRAN), Calle Luisa Fernanda, n° 12, Bj Izq, 28023 Madrid, Spain; 6 Instituto de Rehabilitación Funcional La Salle, Aravaca, 28850 Madrid, Spain

**Keywords:** breast neoplasms, nociceptive pain, neuralgia, chronic pain, cancer pain, central nervous system sensitization, pain catrastrophizing, anxiety, depression

## Abstract

**Simple Summary:**

Persistent pain after breast cancer treatment is still under research due to its complex and multifactorial underlying pathogenesis, including phycological factors. Further research is needed to elucidate more information about the factors that cause and perpetuate this pain. Thus, this study defined the influence of psychosocial and psychological factors on breast cancer survivors who report pain and those who do not. The psychosocial factors assessed were those that are associated with a central sensitization process, and the psychological factors were pain catastrophizing, fear of movement, anxiety and depression. Hence, the psychosocial symptom clusters were identified related to the clinical features of pain or to not reporting pain, which may encourage health clinicians to establish a customized biopsychosocial model focused on the management of pain-catastrophizing thoughts and fear of movement. Furthermore, anxiety and depression should be detected early by health professionals and referred to psychologists to be managed.

**Abstract:**

The frequency of a high Central Sensitization Inventory (CSI) total score and the prevalence of pain have already been established among breast cancer survivors (BCS). However, the psychological factors’ influence based on the clinical features of pain is still unknown, as well as BCS characteristics with no pain. Thus, our main aim was to evaluate the presence of a high CSI total score in BCS with pain and compare it with BCS without pain and to evaluate the influence of psychosocial factors. A cross-sectional comparative study was designed to compare BCS with nociceptive pain (*n* = 19), pain with neuropathic features (*n* = 19) or no pain (*n* = 19), classified by the Leeds Assessment of Neuropathic Symptoms and Signs (LANSS). CSI, pain catastrophizing, fear of movement, anxiety and depression symptoms were analyzed and compared among the three groups. The CSI total score was higher in both BCS pain groups compared to BCS without pain, but there were no statistical differences between the pain groups. The same observation was made when comparing pain catastrophizing. The neuropathic feature group showed greater levels of fear of movement, anxiety and depression compared to the no pain group. Thus, CS-psychosocial associated comorbidities and pain-catastrophizing thoughts were more prevalent among BCS with pain, regardless of the clinical features of pain. BCS with neuropathic pain features showed greater psychological disturbances.

## 1. Introduction

Breast cancer (BC) is the main cause of female cancer in Europe. BC represents 28.8% of female cancers and is the second leading cause of cancer death among women [1]. Curative management for BC involves a multimodality treatment, including breast surgery, radiotherapy and adjuvant/neoadjuvant systemic treatment (cytotoxic chemotherapy, endocrine treatment and biological agents) [2]. As evidence of increasing treatment success and thanks to early diagnosis, the survival rate of women with BC has increased: 87% and 82% at 5 and 10 years after BC diagnosis, respectively [3,4]. Despite this encouraging rate, 21.8% of breast cancer survivors (BCS) report persistent pain after breast cancer treatment (PPBCT), severe pain being more common at 5 years than moderate pain (10.3% and 8.7%, respectively) [5].

When pain becomes persistent, it can be classified as nociceptive, neuropathic, resulting from a central sensitization (CS) process or mixed pain [6,7]. Nociceptive pain has been defined as “pain that arises from actual or threatened damage to non-neural tissue and is due to the activation of nociceptors”. However, when pain is “caused by a lesion or disease of the somatosensory nervous system”, it is named neuropathic pain. Pain elicited by CS processes, called nociplastic pain, is described as “pain that arises from altered nociception despite no clear evidence of actual or threatened tissue damage causing the activation of peripheral nociceptors or evidence for disease or lesion of the somatosensory system causing the pain” [8]. PPBCT can present neuropathic, nociceptive and CS features, with the neuropathic pain component being the most frequent (61.5%) [9].

PPBCT can be triggered by multifactorial causes. These factors include not only those that lead to tissue damage, such as surgery or adjuvant treatment, but also personal factors, such as psychological or demographic conditions. These personal factors can pose a potential risk of having PPBCT [9,10,11]. Regarding the former tissue damage factors, axillary-located nerve manipulation or laceration can occur during axillary surgery. As a result, intercostobrachial, medial brachial cutaneous, thoracodorsal and long thoracic nerves can pose a potential source of neuropathic pain [12]. Moreover, BC medical treatments can lead to various consequences that cause pain [13]. Following radiation therapy, a neural laceration or microvascular neural blockage can occur, increasing mechanosensitivity [14]. Regarding chemotherapy effects, peripheral neuropathy can be induced, especially due to axoplasmic damage. Neuropathy-induced chemotherapy is likely to be a pure sensory symmetrical neuropathy. However, motor fiber damage can lead to motor neuropathy [15]. Hence, chemotherapy, as well as hormone therapy, can lead to sensitivity alteration of the peripheral nerves and even affect central level pain processing [9,16]. CS can appear when the nervous system is exposed to threatening inputs, leading to pain hypersensitivity (i.e., secondary hyperalgesia and allodynia). CS mechanisms could be involved in PPBCT. Indeed, BCS showed primary and secondary hyperalgesia, temporal summation disturbance 6 months after surgery and after having completed radiation and chemotherapy treatments [17,18,19].

Regarding the latter psychological PPBCT-involved factors, a positive psychological attitude is correlated with being less likely to experience PPBCT. Hence, those BCS prone to anxiety, depression, catastrophism, Kinesiophobia or poor quality of sleep are at risk of PPBCT [10,11].

To the best of our knowledge, no previous studies have investigated whether there are differences in CS-psychosocial associated comorbidities based on the clinical features of pain or on not reporting pain. Thus, our main objective was to evaluate the presence of a high CSI total score related to CS-psychosocial comorbidities based on pain with nociceptive or neuropathic features in BCS compared with BCS without pain. The secondary objective was to evaluate the differences in psychological variables, such as pain catastrophizing, fear of movement, anxiety and depression, among BCS with nociceptive pain, with pain with neuropathic features and without pain. Moreover, we aimed to discover the correlations among CS-psychosocial comorbidities assessed by CSI and psychological variables within each group in order to study the potential psychosocial symptom clusters in BCS.

## 2. Materials and Methods

### 2.1. Study Design

A cross-sectional study design was used to assess the differences in CSI total score related to CS-psychosocial associated comorbidities and psychological variables of breast cancer survivors with nociceptive pain, pain with neuropathic features and no pain. The trial was conducted in accordance with the Strengthening the Reporting of Observational Studies in Epidemiology statement [20]. All procedures were approved by the Human Research Ethics Committee of the La Salle University Center for Advanced Studies (CSEULS-PI-009/2019). All participants granted their written informed consent prior to inclusion and were provided with an explanation of the study procedures, which were planned under the ethical standards of the Helsinki Declaration.

### 2.2. Participants

A total of 57 women who underwent breast cancer treatment were recruited from the Torrejón University Hospital, Madrid (Spain), by non-probabilistic convenience sampling. The sample was collected between October 2020 and June 2021 using the following inclusion criteria: (a) women who had undergone unilateral breast cancer surgery at least one-and-a-half years previously; (b) women with axillary surgery using the selective sentinel node biopsy technique or lymphadenectomy; and (c) participants had to have completed their adjuvant radiotherapy and/or chemotherapy treatment. Those who were receiving hormonal therapy could be included in the study. The exclusion criteria were as follows: (a) women who presented bilateral breast cancer or locoregional relapse and who were receiving adjuvant chemotherapy or radiotherapy; (b) women who reported pain prior to surgery; (c) women diagnosed with peripheral neuropathy before or after BC treatment; (d) women who were not Spanish speaking; (e) women who presented cognitive diseases; and (f) women who had undergone a cancer process in another organ.

### 2.3. Procedure

After giving their written informed consent, the participants completed a sociodemographic questionnaire that collected information on sex, date of birth, marital status, educational level and professional activity and were asked about their clinical and surgical history related to the cancer process (type of surgery, treatments, etc.). The participants then completed the self-report measures to determine their pain catastrophism, the level of fear of movement, level of anxiety and depression and the Leeds Assessment of Neuropathic Symptoms and Signs (LANSS) pain scale. The sequence of administration of the self-report questionnaires was chosen randomly for each participant to control for possible systematic bias. Patients who obtained a LANSS score ≥12 were included in the group of pain with neuropathic features, while those with a LANSS score <12 were included in the group with nociceptive pain [21]; those women who did not experience pain formed a third group. Women with a LANSS score ≥12 were classified as having pain with neuropathic features, not as having neuropathic pain, since confirmatory diagnostic tests of somatosensory lesion or disease are needed to diagnose neuropathic pain [22,23]. The LANSS questionnaire has good discriminant and construct validity. The Spanish validation has an internal consistency by Cronbach’s coefficient of between 0.68 and 0.71 and good inter-rater agreement (κ = 0.70) and intra-class correlation coefficients of between 0.77 and 0.92 [24].

### 2.4. Outcome Measures

#### 2.4.1. Primary Outcomes

- CSI total score

The CSI is a self-report measure used to assess the severity of symptoms that may be related to a possible CS process, regardless of a specific etiology, with good psychometric characteristics [25,26,27]. Thus, research studies have used the CSI questionnaire to evaluate symptoms compatible with a CS process [6,9,26,28,29]. However, CSI is not a tool for the diagnosis of CS [26,30], since CSI did not show associations with psychophysical tests, such as pain pressure thresholds [31,32,33,34,35], conditioned pain modulation [32,34,35] and temporal summation [33,34,35]. However, CSI had shown a positive correlation with psychosocial factors in several populations [31,32,33,34,35]. Hence, CSI is associated with general distress, probably enhanced by a CS process [32], rather than with identifying a CS process [33].

The CSI questionnaire consists of two sets of questions: one set of 25 questions with a maximum score of 100 and the other with different medical diagnoses. A high CSI total score is considered when it is ≥40 points [6,25]. The CSI Spanish validation has been demonstrated to be a psychometrically strong measure for assessing CS symptoms in BCS based on internal consistency, test–retest reliability and structural validity. The internal consistency was high (α = 0.91), as well as the test–retest reliability (ICC 2.1 = 0.95) [36].

#### 2.4.2. Secondary Outcomes

- Anxiety and depression

Anxiety and depression levels were assessed using the Hospital Anxiety and Depression Scale (HADS). The scale consists of a 7-item anxiety subscale and a 7-item depression subscale. Each item scores on a 4-point Likert scale, giving maximum subscale scores of up to 21 points each for depression and anxiety [37]. The HADS presented an internal consistency (Cronbach’s alpha) from 0.80 to 0.93 for the anxiety and 0.81 to 0.90 for the depression subscales [38].

- Pain catastrophizing

The Spanish version of the Pain Catastrophizing Scale (PCS) assesses the degree of pain catastrophizing [39]. It is composed of 13 items with a numeric value between 0 (not at all) and 4 (all the time), with a maximum score of 52 points (higher scores indicate more catastrophizing). It has 3 subscales: rumination (range = 0–16), magnification (range = 0–12) and helplessness (range = 0–24). Higher scores indicate greater pain catastrophizing [40,41,42]. The PCS is a reliable and valid measure of pain catastrophizing (Cronbach’s alpha = 0.75–0.95) [43,44].

- Fear of movement

Fear of movement was assessed using the 11-item Spanish version of the Tampa Scale of Kinesiophobia, which has a Cronbach’s alpha of 0.78 [45]. The final score can range between 11 and 44 points, with higher scores indicating greater perceived fear of movement [45].

### 2.5. Sample Size

We conducted a pilot study to determine the effect size between breast cancer survivors with nociceptive pain, pain with neuropathic features or no pain, using the CSI total score related to CS-psychosocial associated comorbidities. The pilot study included 8 patients from each group and obtained an f (Cohen’s f statistic) of 0.43 [46]. The sample size was estimated with G*Power for Windows from the University of Düsseldorf, Germany [47]. A one-way analysis of variance (ANOVA) was employed to detect differences between groups for the CSI total score, which was the only one in which statistically significant differences were obtained. Moreover, we used an alpha error level of 0.05, a statistical power of 80% (1-B error) and an effect size of 0.43. The minimum sample size for statistical significance based on this calculation was *n* = 19 per group. No losses were assumed due to the cross-sectional nature of the study. Thus, a total sample size of 57 patients (19 patients with nociceptive pain, 19 patients with pain with neuropathic features and 19 patients without pain) was estimated to ensure reliability.

### 2.6. Data Analysis

The sociodemographic and clinical variables of the participants were analyzed. The data were summarized using frequency counts, descriptive statistics, summary tables and figures. The data analysis was performed using the Statistics Package for the Social Sciences (SPSS 27.00, IBM Inc., Armonk, NY, USA). The categorical variables are shown as frequency and percentage. The quantitative results of the study are represented by descriptive statistics (CI, mean and standard deviation). A normality analysis was performed using the Shapiro–Wilk test, and all variables followed a normal distribution [48,49].

Multiple comparison tests of outcome variables were used for the three groups in the study. Cohen’s d effect sizes were calculated for a post hoc analysis by Bonferroni correction. According to Cohen’s method, the magnitude of the effect was classified as small (0.20 to 0.49), medium (0.50 to 0.79) or large (0.80) [50].

A one-way ANOVA was used to analyze numerical variables among the 3 groups (sociodemographic variables, CSI total score, pain catastrophism, fear of movement, anxiety and depression). A chi-squared test with residual analysis was used to compare categorical variables [50,51].

We examined the associations between all variables using Pearson’s correlation coefficient. A Pearson’s correlation coefficient >0.60, 0.30 to 0.60 and <0.30 indicated high, medium and low correlations, respectively [49].

## 3. Results

A total of 57 participants completed the study (19 patients with neuropathic pain, 19 patients with nociceptive pain and 19 patients without pain). Table 1 shows the sociodemographic characteristics of the study participants.

Considering the clinical characteristics in relation to the cancer process, the results only show statistically significant differences between groups in terms of treatment with chemotherapy. The symptomatic groups have a higher percentage of patients treated with chemotherapy and hormone therapy. The group with pain with neuropathic characteristics has a higher percentage of patients treated with chemotherapy (Table 2).

### 3.1. Primary Variable

**Figure 1:**
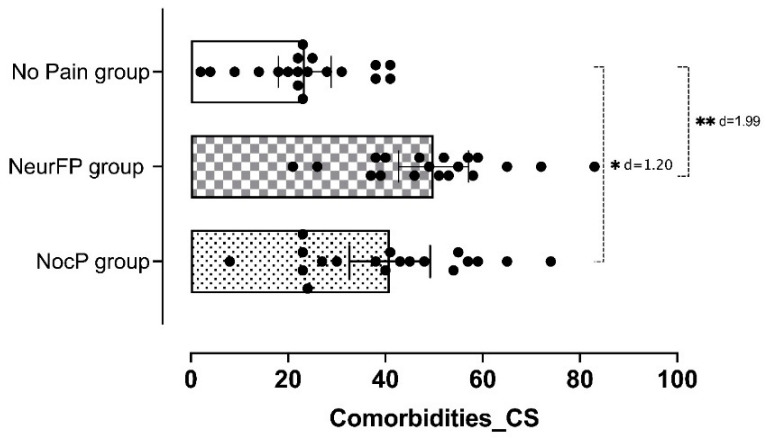
Comparison of CS-psychosocial associated comorbidities assessed by CSI between groups. CS: Central sensitization; CSI: Central Sensitization Inventory; NocP group: nociceptive pain group; NeurFP group: neuropathic features pain group. * *p* < 0.05; ** *p* < 0.001.

**Figure 2:**
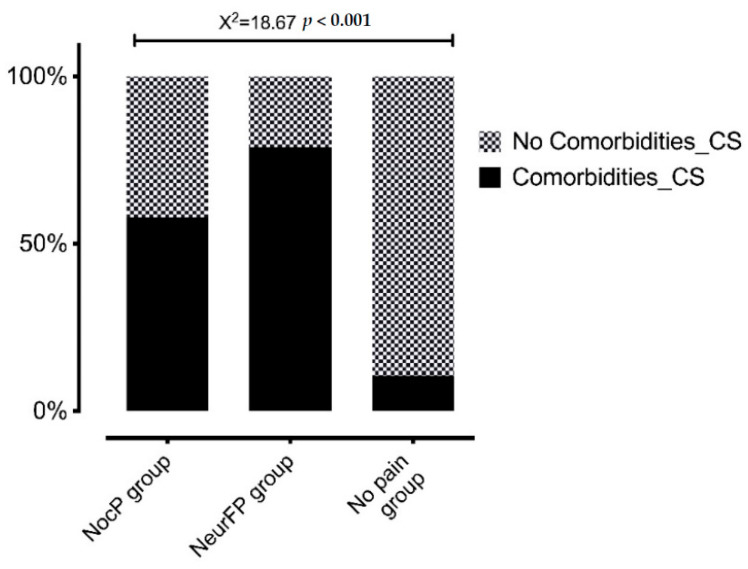
Percentage of patients in each group presenting CS-psychosocial associated comorbidities assessed by CSI. CS: Central sensitization; NocP group: nociceptive pain group; NeurFP group: neuropathic features pain group.

Statistically significant differences were observed in the CSI total score between patients who presented pain with any clinical features and those who did not (F = 15.9; *p* < 0.001). Figure 1 shows the CSI total score differences between groups and Figure 2 the percentage of patients within each group who passed the CSI cut-off point (≥40 points).

An ANOVA revealed significant CSI total score differences between the patients who presented nociceptive pain and those who did not have pain (*p* = 0.002; d = 1.20) and between patients who presented neuropathic pain and those who did not have pain for the same variable (*p* < 0.001; d = 1.99). Table 3 shows the intergroup comparison (mean differences).

### 3.2. Secondary Variables

Regarding the secondary variables, the results showed significant differences in pain catastrophizing between the nociceptive pain group and the group without pain (*p* = 0.028; d = 0.96) and between the neuropathic pain feature group and the group without pain (*p* = 0.006; d = 1.12). Statistically significant differences were also shown for the variables of fear of movement (*p* = 0.016; d = 0.89), anxiety (*p* < 0.001; d = 1.41) and depression (*p* = 0.015; d = 0.99) between the neuropathic pain feature group and the group of patients without pain (Table 3).

### 3.3. Correlation Analysis

Table 4 shows the results of the correlation analysis for the symptomatic groups. For the nociceptive pain group, the strongest positive correlations were between the CSI total score and pain catastrophizing (r = 0.465; *p* < 0.01) and between pain catastrophizing and depression (r = 0.763; *p* < 0.01). In contrast, the neuropathic feature pain group did not present any correlation between the main variable of CSI total score and the remaining psychological variables. Two of the strongest correlations for this group were between pain catastrophizing and fear of movement (r = 0.841; *p* < 0.01), as well as between fear of movement and anxiety (r = 0.688; *p* < 0.01).

### 3.4. Psychosocial Symptom Cluster

This study showed a possible psychosocial symptom cluster related to clinical features of PPBCT or to not reporting pain, as follows:

BCS with nociceptive pain seemed to be related to CS-psychosocial associated comorbidities (CSI total score = 40.9 ± 17.2), pain-catastrophizing thoughts (PCS = 18.7 ± 14.7) and fear of movement (TSK-11 = 22.8 ± 6.9). Pain-catastrophizing thoughts were strongly correlated with CS-psychosocial associated comorbidities and with depression symptoms.

BCS with pain with neuropathic features seemed to be related to CS-psychosocial associated comorbidities (CSI total score = 49.8 ± 14.9), pain-catastrophizing thoughts (PCS = 21.1 ± 15.4), fear of movement (TSK-11 = 28.6 ± 8.0) and anxiety symptoms (HADS anxiety subscale = 9.4 ± 4.8). Fear of movement was strongly associated with pain-catastrophizing thoughts and anxiety symptoms.

BCS without pain only presented fear of movement (TSK-11 = 21.5 ± 7.9) as a psychological disturbance.

## 4. Discussion

In this study, LANSS was used to classify BCS regarding their pain clinical features, whereas a previous study did so through a clinical algorithm [9]. Our purpose was to classify the participants’ clinical features of pain rather than the etiology of pain. LANSS is a robust tool for identifying patients whose pain arises from neuropathic mechanisms [21], with 85% sensitivity and 80% specificity [52]. Hence, it has commonly been used as a guideline for classifying patients with pain with neuropathic pain features vs. nociceptive pain in other populations [53,54].

The CSI is a questionnaire that helps identify symptoms mediated by CS mechanisms, regardless of a specific etiology, with good psychometric characteristics [25,26,27]. The cut-off of 40 points on the CSI leads to detection of over 82% of patients with CS; however, the false-positive odds are relatively high [6]. Thus, somatosensory exploration is needed to objectively identify CS pain [6], for which quantitative sensory testing (QST) is the gold standard [55,56]. This standardized method is widely used in research to detect CS, but the length of time and the cost of the equipment required impede its use in current practice [6,57]. Thus, QST is often performed partially [31,32,33,34,35].

Despite its limited ability to identify CS pain due to the scarce correlation with psychophysical tests [31,32,33,34,35], the CSI reliably detects CS-psychosocial related symptoms [31,33,34,35,58]. Thus, our objective was to determine the presence of CS-psychosocial associated comorbidities using the CSI questionnaire among BCS with nociceptive pain, with pain with neuropathic features and without pain, and to determine its correlation with phycological variables assessed by PCS, TSK-11 and HADS questionnaires.

The present study found CS-psychosocial associated comorbidities in BCS with pain with a high CSI total score, as previously reported [9,19,59,60,61]. Furthermore, that higher CSI total score found in patients with pain was statistically different compared with patients without pain, regardless of their clinical features of pain. In addition, in our study, there were significantly more frequent higher CSI total scores in patients with neuropathic features (78.9%) or nociceptive pain (57.9%) compared to BC pain without pain (10.5%). Leysen et al. also showed CSI results based on the clinical pain features. They classified BCS pain as nociceptive, neuropathic and CS pain by using a clinical algorithm where the mandatory psychophysical tests (QST) to diagnose neuropathic pain—despite performing a physical exploration through the DN4 questionnaire—[22,23] or CS pain [55,56] are lacking [9]. In this regard, we only determined the clinical features of pain—despite performing a physical exploration using the LANSS questionnaire—and the CS-psychosocial associated comorbidities assessed by CSI, since we, likewise, did not perform psychophysical tests (QST). In addition, BCS without pain were not included in their study, and apart from CS-psychosocial associated comorbidities evaluated by CSI, psychological comorbidities were not assessed [9], as the present study had done. Unlike the rest of the BCS studies, Hurth et al. showed CSI results in BCS without pain. However, Hurth et al. did not classify the BCS clinical features of pain [59]; thus, the CSI differences based on clinical pain features were not provided.

The local and widespread somatosensory exploration that should accompany CSI to confirm CS pain has only been performed by De Groef et al. They found the pressure pain thresholds (PPTs) decreased on the affected upper limb [61]. However, other local and widespread somatosensory findings, such as thermal thresholds, von Frey filament-induced touch allodynia, pinprick-induced mechanical hyperalgesia and temporal summation (wind-up phenomenon), are needed to confirm CS pain [55]. Along these lines, the current studies show the somatosensory profiles among BCS with pain, but the CSI is not addressed [17,18,62,63,64].

In terms of the influence of pain catastrophizing on the CSI, both pain groups showed pain catastrophizing compared with the no pain group, given that the PCS score was higher than 15 points in both pain groups. Moreover, there was a positive correlation between a high CSI total score and pain catastrophizing. This result is in line with Manfuku et al., 2019, and de Groef et al., 2018, who concluded that this correlation is associated with the development and/or maintenance of persistent pain in these patients [19,61]. Kanzawa-Lee et al. also found a negative correlation between bilateral-trapezius PPTs and pain catastrophizing [62], which concurs with the CSI-psychosocial associated comorbidities and pain catastrophizing correlation. Moreover, Edwards et al. showed that pain catastrophizing could mediate the association between PPBCT and evoked pain sensitivity in BCS, as well as between the expectancies and pain facilitatory processes [65,66]. Pain catastrophizing has also been shown to predict chronic pain severity in patients with lung cancer [67]. Pain catastrophizing is a maladaptive pain cognition that increases the impact of pain due to the magnification of pain severity and sensitivity and the central interaction with other symptoms, such as anxiety and fatigue [68,69]. This agrees with the correlations that pain catastrophizing showed with anxiety, depression and fear of movement in this study.

Concerning the fear of movement, all three groups presented it, but it was significantly higher in the group with pain with neuropathic features compared with the no pain group. However, no differences were found between the pain groups or between the nociceptive group and the no pain group. This result could imply that the feelings of fragility in BCS are more likely to be linked to suffering from pain when it is associated with neuropathic features rather than the cancer’s emotional impact. Moreover, fear of movement showed a positive correlation with pain catastrophizing, as well as with anxiety and depression, as Can et al. concluded. In addition to these emotional pain management associations, fear of movement increases the likelihood of lymphedema and reduces upper limb functional capacities [70].

Considering the suggested ≥8 points as a screening threshold for HADS depression/anxiety, anxiety was detected in the group with pain with neuropathic features. The only significant difference among the groups was found between the group with pain with neuropathic features and the no pain group, showing significantly higher levels of anxiety and depression [71]. Likewise, Park et al. found a prevalence index of 44% for anxiety and 20% for depression by means of HADS among young women with metastatic BC [71]. Other studies also found high levels of anxiety and depression, although the HADS was not used [65,72].

Unlike our study, Hurth et al. recently reported a moderate to strong correlation between CSI and the HADS anxiety (r = 0.68) and depression (r = 0.67) scales, which is consistent with the factor of “emotional distress” and with previous studies [27,73].

Our findings related to greater fear of movement as well as greater anxiety and depression levels in the neuropathic feature pain group revealed the emotional impact of neuropathic pain. Pain with neuropathic features can arise due to BC treatment, regardless of the type of axillary intervention [12]. Moreover, it can occur because of chemotherapy-induced peripheral neuropathy, given that it is one of the most frequent toxicities associated with taxane use as a curative early stage BC treatment. The pain usually arises during treatment and tends to persist for many years [74].

Persistent post-surgery neuropathic pain triggers mechanisms that underlie a complex dynamic process. Excessive peripheral and central neural inputs, such as dysregulated sensory neural pathways, dysregulated activity of specific neurotransmitters and cognitive and emotional neural circuits, and the balance between degenerative and regenerative neural events can lead to CS and the consequent persistent post-surgery neuropathic pain [75].

### Limitations

This study has several limitations. First, it did not evaluate somatosensory alterations by means of sensory quantitative tests. It will be necessary in future studies to perform this evaluation together with the psychosocial evaluation performed in the present study to identify whether there is an underlying CS process in BCS with PPBCT. Another important limitation of this research is the lack of evaluation of physical activity, which can directly influence the pain experience of each patient. Furthermore, we did not assess whether the patients were taking medication and the type of medication, which could also have influenced the results.

Finally, the results of the present study should be interpreted with caution, given it is a cross-sectional study; thus, causal relationships cannot be established. However, we can infer that both BCS with nociceptive pain and BCS with pain with neuropathic features are likely to be related to greater CS-psychosocial associated comorbidities and catastrophizing thoughts than BCS without pain. Moreover, BCS with pain with neuropathic features are also more likely to show further levels of fear of movement, anxiety and depression compared to BCS without pain. We suggest conducting a future longitudinal study where chemotherapy and hormonotherapy are independent variables. Thus, these cognitive-emotional characteristics would be evaluated prior to adjuvant treatment administration to determine whether the persistence and the psychosocial impact of pain differed as a function of the type of adjuvant treatment.

## 5. Conclusions

CS-psychosocial associated comorbidities assessed by CSI are more prevalent among BCS with pain, regardless of the clinical features of pain, as occurs with pain-catastrophizing thoughts. BCS with pain with neuropathic features show greater fear of movement as well as anxiety and depression than BCS without pain. A biopsychosocial model focused on the avoidance of pain-catastrophizing thoughts might be needed as a guideline for the entire health community to effectively manage patients with BC. Further longitudinal studies are suggested to verify our results.

## Figures and Tables

**Table 1:** Descriptive statistics for demographic outcomes.

Measures	NocP Group (*n* = 19)	NeurFP Group (*n* = 19)	No Pain Group (*n* = 19)	*p* Value
Age a	57.9 ± 11.5	56.1 ± 6.3	63.1 ± 8.6	0.089
Height a	1.60 ± 0.06	1.60 ± 0.06	1.59 ± 0.08	0.063
Weight a	67.7 ± 7.3	68.7 ± 19.9	68.9 ± 11.4	0.088
Marital Status b				0.538
Single (%)	14 (73.7)	13 (68.4)	14 (73.7)	
Married (%)	1 (5.3)	4 (21.1)	1 (5.3)	
Widow (%)	2 (10.5)	0 (-)	2 (10.5)	
Divorced (%)	2 (10.5)	2 (10.5)	2 (10.5)	
Professional Status b				0.002 *
Working (%)	4 (21.1)	9 (47.3)	9 (47.3)	
Not working (%)	11 (57.9)	3 (15.8)	4 (21.1)	
Retired (%)	3 (15.8)	1 (5.3)	6 (31.6)	
Sick leave (%)	1 (5.3)	6 (31.6)	0 (-)	

NocP group: nociceptive pain group; NeurFP group: neuropathic features pain group. a One-way analysis of variance *p* value. b Chi-square test. * *p* < 0.05.

**Table 2:** Descriptive statistics for clinical variables and medical treatments.

Measures	NocP Group (*n* = 19)	NeurFP Group (*n* = 19)	No Pain Group (*n* = 19)	*p* Value
CS-psychosocial associated comorbidities by CSI				<0.001
Yes (%)	11 (57.9)	15 (78.9)	2 (10.5)	
No (%)	8 (42.1)	4 (21.2)	17 (89.5)	
Axillary Surgery				0.523
Selective sentinel node biopsy (%)	10 (52.6)	11 (57.9)	13 (68.4)	
Lymphadenectomy (%)	6 (31.6)	6 (31.6)	6 (31.6)	
Both surgeries (%)	3 (15.8)	2 (10.5)	0 (-)	
Type of breast surgery b				0.878
Tumorectomy (%)	4 (21.1)	7 (36.8)	6 (31.6)	
Mastectomy (%)	6 (31.6)	5 (26.3)	5 (26.3)	
Quadrantectomy (%)	9 (47.3)	7 (36.8)	8 (42.1)	
Lymphedema b				0.151
Yes (%)	7 (36.8)	4 (21.1)	2 (10.5)	
No (%)	12 (63.2)	15 (78.9)	17 (89.5)	
Chemotherapy b				0.017 *
Yes (%)	16 (84.2)	17 (89.5)	10 (52.7)	
No (%)	3 (15.8)	2 (10.5)	9 (47.3)	
Hormonotherapy b				0.004 *
Yes (%)	9 (47.3)	16 (84.2)	6 (31.6)	
No (%)	10 (52.7)	3 (15.8)	13 (68.4)	
Radiotherapy b				0.803
Yes (%)	18 (94.7)	17 (89.5)	17 (89.5)	
No (%)	1 (5.3)	2 (10.5)	2 (10.5)	

NocP group: nociceptive pain group; NeurFP group: neuropathic features pain group. CS: Central sensitization; CSI: Central Sensitization Inventory. b Chi-square test. * *p* < 0.05.

**Table 3:** Descriptive and multiple comparisons of psychological variables.

Measures	NocP Group (*n* = 19)	NeurFP Group (*n* = 19)	No Pain Group (*n* = 19)	Difference of Means (95% CI); Effect Size (d); NocP Group vs. NeurFP Group NocP Group vs. No Pain Group NeurFP Group vs. No Pain Group
CS-psychosocial associated comorbidities by CSI	40.9 ± 17.2	49.8 ± 14.9	23.4 ± 11.3	−8.94 (−20.7; 2.84) d = −0.55 17.47 * (5.69; 29.26) d = 1.20 26.42 ** (14.63; 38.21) d = 1.99
Pain Catastrophizing	18.7 ± 14.7	21.1 ± 15.4	7.2 ± 8.4	−2.36 (−12.9; 8.21); d = −0.15 11.52 * (0.95; 22.10); d = 0.96 13.89 * (3.32; 24.47); d = 1.12
Rumination subscale	6.2 ± 5.4	7.2 ± 5.1	2.8 ± 3.5	−1.05 (−4.85; 2.75); d = −0.19 3.36 (−0.43; 3.17); d = 0.74 4.42 * (0.62; 8.22); d = 1.01
Magnification subscale	4.5 ± 3.2	4.8 ± 4.1	2.1 ± 2.1	−0.26 (−2.88; 2.35); d = −0.08 2.47 (−0.14; 5.09); d = 0.89 2.73 * (0.12; 5.35); d = 0.83
Helplessness subscale	14.1 ± 12.2	14.3 ± 12.1	3.8 ± 6.4	−0.21 (−8.69; 8.27); d = −0.02 10.31 * (1.83; 18.80); d = 1.06 10.52 * (2.04; 19.01); d = 1.08
Fear of movement	22.8 ± 6.9	28.6 ± 8.0	21.5 ± 7.9	−5.84 (−11.96; 0.27); d = −0.78 1.31 (−4.80; 7.43); d = 0.17 7.15 * (1.04; 13.27); d = 0.89
Fear of PA subscale	13.9 ± 4.7	17.4 ± 5.7	14.1 ± 5.3	−3.47 (−7.78; 0.83); d = −0.67 −0.21 (−4.51; 4.09); d = −0.04 3.26 (−1.04; 7.57); d = 0.60
Fear of harm subscale	8.9 ± 3.2	11.3 ± 3.5	7.4 ± 3.9	−2.36 (−5.20; 0.46); d = −0.72 1.52 (−1.31; 4.36); d = 0.42 3.89 * (1.06; 6.73); d = 1.05
Anxiety	6.4 ± 3.9	9.4 ± 4.8	3.7 ± 3.1	−3.00 (−6.19; 0.19); d = −0.69 2.68 (−0.51; 5.87); d = 0.77 5.68 ** (2.49; 8.87); d = 1.41
Depression	4.4 ± 3.8	6.1 ± 4.2	2.5 ± 3.0	−1.68 (−4.66; 1.29); d = −0.42 1.84 (−1.13; 54.81); d = 0.55 3.52 * (0.55; 6.50); d = 0.99

Values are presented as mean ± standard deviation. NocP group: nociceptive pain group; NeurFP group: neuropathic features pain group; CS. Central sensitization; CSI: Central Sensitization Inventory; PA: physical activity. * *p* < 0.05; ** *p* < 0.001.

**Table 4:** Correlation analysis examining the bivariate relationships between the psychological variables and the sensorimotor variables.

		Comorbidities_CS	Pain Catastrophizing	Rumination Subscale	Magnification Subscale	Helplessness Subscale	Fear of Movement	Fear of PA Subscale	Fear of Harm Subscale	Anxiety	Depression
CS-psychosocial associated comorbidities by CSI	NocP group	1.00	0.465 *	0.483 *	0.383	0.435	0.382	0.373	0.269	0.224	0.275
	NeurFP group	1.00	0.299	0.365	0.415	0.280	0.269	0.382	−0.034	0.379	0.355
Pain Catastrophizing	NocP group		1.00	0.968 **	0.926 **	0.945 **	0.736 **	0.538 *	0.789 **	0.509 *	0.763 **
	NeurFP group		1.00	0.956 **	0.923 **	0.909 **	0.841 **	0.760 **	0.629 **	0.829 **	0.371
Rumination subscale	NocP group			1.00	0.885 **	0.890 **	0.678 **	0.487 *	0.740 **	0.503 *	0.777 **
	NeurFP group			1.00	0.890 **	0.802 **	0.795 **	0.721 **	0.592 **	0.828 **	0.377
Magnification subscale	NocP group				1.00	0.786 **	0.604 **	0.453	0.630 **	0.490 *	0.683 **
	NeurFP group				1.00	0.789 **	0.776 **	0.755 **	0.492 *	0.778 **	0.332
Helplessness subscale	NocP group					1.00	0.738 **	0.549 *	0.777 **	0.487 *	0.760 **
	NeurFP group					1.00	0.770 **	0.727 **	0.524 *	0.799 **	0.508 *
Fear of movement	NocP group					.	1.00	0.915 **	0.797 **	0.599 **	0.555 *
	NeurFP group						1.00	0.915 **	0.730 **	0.688 **	0.311
Fear of PA subscale	NocP group							1.00	0.485 *	0.482 *	0.360
	NeurFP group							1.00	0.391	0.674 **	0.362
Fear of harm subscale	NocP group								1.00	0.577 **	0.665 **
	NeurFP group								1.00	0.426	0.096
Anxiety	NocP group									1.00	0.699 **
	NeurFP group									1.00	0.640 **
Depression	NocP group										1.00
	NeurFP group										1.00

NocP group: nociceptive pain group; NeurFP group: neuropathic features pain group; CS: central sensitization; CSI: Central Sensitization Inventory; PA: physical activity. * *p* < 0.05; ** *p* < 0.001.

## Data Availability

Not applicable.
